# Dysphagia as a risk factor for mortality in Niemann-Pick disease type C: systematic literature review and evidence from studies with miglustat

**DOI:** 10.1186/1750-1172-7-76

**Published:** 2012-10-06

**Authors:** Mark Walterfang, Yin-Hsiu Chien, Jackie Imrie, Derren Rushton, Danielle Schubiger, Marc C Patterson

**Affiliations:** 1 Royal Melbourne Hospital and Melbourne Neuropsychiatry Centre, Melbourne 3050, Australia; 2 Departments of Paediatrics and Medical Genetics, National Taiwan University Hospital, Taipei, Taiwan; 3 University of Manchester, Manchester, UK; 4 Actelion Pharmaceuticals Pty Ltd, New South Wales, Australia; 5 Mayo Clinic, Rochester MN, USA

**Keywords:** Niemann-Pick disease type C, Dysphagia, Mortality, Swallowing, Pneumonia, Aspiration, Miglustat.

## Abstract

Niemann-Pick disease type C (NP-C) is a rare neurovisceral disease characterised by progressive neurological deterioration and premature death, and has an estimated birth incidence of 1:120,000. Mutations in the *NPC1* gene (in 95% of cases) and the *NPC2* gene (in approximately 4% of cases) give rise to impaired intracellular lipid metabolism in a number of tissues, including the brain. Typical neurological manifestations include vertical supranuclear gaze palsy, saccadic eye movement abnormalities, cerebellar ataxia, dystonia, dysmetria, dysphagia and dysarthria. Oropharyngeal dysphagia can be particularly problematic as it can often lead to food or fluid aspiration and subsequent pneumonia. Epidemiological data suggest that bronchopneumonia subsequent to food or fluid aspiration is a major cause of mortality in NP-C and other neurodegenerative disorders. These findings indicate that a therapy capable of improving or stabilising swallowing function might reduce the risk of aspiration pneumonia, and could have a positive impact on patient survival. Miglustat, currently the only approved disease-specific therapy for NP-C in children and adults, has been shown to stabilise key neurological manifestations in NP-C, including dysphagia. In this article we present findings from a systematic literature review of published data on bronchopneumonia/aspiration pneumonia as a cause of death, and on the occurrence of dysphagia in NP-C and other neurodegenerative diseases. We then examine the potential links between dysphagia, aspiration, pneumonia and mortality with a view to assessing the possible effect of miglustat on patient lifespan.

## Introduction

Niemann-Pick disease type C (NP-C) is a rare neurovisceral disease characterised by progressive neurological deterioration and premature death, and has an estimated birth incidence of 1:120,000 [1,2]. It is caused by the autosomal recessive inheritance of mutations in either of the two genes, *NPC1* (in approximately 95% of cases) or *NPC2* (in approximately 4% of cases) [3-5]. Mutations in either of these genes result in impaired metabolism of endocytosed cholesterol and intracellular accumulation of a number of lipid moieties in various tissues, particularly in the brain [1,6].

Patients with NP-C usually present with one or more neurological signs during childhood [1], although an increasing number of patients with adult onset of neurological manifestations are being diagnosed based on late-onset neurological signs and psychiatric manifestations [7-9]. The age at onset of neurological manifestations has a major influence on the rate of disease progression and prognosis. In general, patients with neurological onset early in life deteriorate faster and have a shorter life expectancy than those with adult onset [1,10-14].

The clinical presentation of NP-C is extremely heterogeneous. Systemic symptoms such as neonatal jaundice and hepatosplenomegaly usually occur early in the course of the disease [1,15]. Typical neurological manifestations include vertical supranuclear gaze palsy (VSGP), saccadic eye movement (SEM) abnormalities, cerebellar ataxia, dystonia, dysmetria, dysarthria and dysphagia [1]. These neurological signs arise at different ages, but invariably progress over time [10,16].

Dysphagia occurs in most NP-C patients at some point in the disease course, ranging in severity from occasional swallowing difficulties to loss of swallowing function necessitating placement of a nasogastric tube or gastrostomy feeding [10]. Dysphagia of neurological origin arises primarily from impairment in the oral and pharyngeal phases as opposed to the later oesophageal phases of swallowing, and as such is defined as oropharyngeal dysphagia [17]. Oropharyngeal dysphagia disrupts feeding but, more significantly, it increases the risk of aspiration. In NP-C, it is contributed to by bulbar motor dysfunction, dystonia, and reduced sensation. Patients with more severe neurological involvement generally have more severe dysphagia, and worsening neurological involvement correlates with a higher risk of aspirating food or fluid [18]. It is therefore recommended that patients with impaired swallowing function be closely monitored to avoid serious lung infections secondary to aspiration [15] and to ensure adequate nutrition.

Data from epidemiological studies suggest that bronchopneumonia subsequent to food or fluid aspiration is a major cause of mortality in NP-C and other neurodegenerative disorders. While precise causes of death are not consistently reported in NP-C, two studies have identified bronchopneumonia as the leading cause of death in two separate NP-C patient cohorts, accounting for approximately two-thirds of patients [19,20]. Marik et al. recognised aspiration pneumonia as the leading cause of death among a range of neurodegenerative disorders [21]. Among factors known to contribute to the development of aspiration pneumonia (e.,g., poor oral hygiene, sleep disorders, emesis), dysphagia is considered to contribute by far the greatest risk [22].

Substrate reduction therapy with miglustat (Zavesca®; Actelion Pharmaceuticals) was first approved for treatment of progressive neurological deterioration in children and adults with NP-C in Europe in 2009, and has since been approved in a number of other countries [23]. The primary therapeutic mode of action of miglustat in NP-C is thought to be the reduction of glucosylceramide-based glycosphingolipid synthesis in the CNS, through the reversible inhibition of glucosylceramide synthase [15,24]. In clinical studies miglustat therapy stabilised key neurological manifestations of the disease in adults and children [25-27]. While experience with the use of miglustat in clinical practice settings is increasing [11,18,28-31], published data on long-term clinical outcomes in NP-C patients receiving miglustat remain relatively scarce, owing to the rarity of the disease.

The progressive neurodegenerative nature of NP-C and its typically delayed diagnosis means that disease stabilisation, or a reduced rate of disease progression, are the best attainable goals for long-term disease-specific therapy, given that most patients have a substantial burden of disease by the time of diagnosis [15]. Published data suggest that therapies capable of stabilising/improving swallowing function can reduce the risk of aspiration pneumonia and, potentially, reduce mortality risk [32,33]. In this article we review published information on the most common reported causes of death in NP-C, and evaluate disease factors that might contribute to an increased risk of mortality. We then examine the impact of miglustat on these disease factors to gain insight into the potential effects of this drug on patient lifespan.

## Systematic review methodology

### Epidemiological research

A series of systematic epidemiological literature reviews was conducted using the Embase online database in March 2011, to investigate several disease-related factors. Owing to the rarity of the disease and to limited long-term outcome reporting in NP-C (such as precise causes of death), literature reviews examining disease factors specific to NP-C were conducted in parallel with searches on a range of other neurodegenerative diseases. We compared outcomes data in NP-C with those from disorders with similar motor manifestations (particularly dysphagia), and similar neurodegenerative courses [34,35]. Data from patients with acute stroke were also evaluated because of the frequency of dysphagia following stroke [36]. Searches were performed to investigate the following disease-related factors:

1. Causes of death in NP-C (key terms: NP-C, mortality, cause of death)

2. Causes of death in progressive neurodegenerative disease in general (key terms: mortality, neurodegenerative disease*)

3. The prevalence of dysphagia in neurodegenerative diseases including NP-C (key terms: swallowing, dysphagia, neurodegenerative disease*, NP-C)

4. The association between dysphagia and aspiration pneumonia (key terms: swallowing, dysphagia, aspiration pneumonia, neurodegenerative disease*, NP-C, stroke, traumatic brain injury)

5. The association between aspiration pneumonia and mortality (key terms: death, cause of death, mortality, aspiration pneumonia, neurodegenerative disease*, NP-C, stroke, traumatic brain injury).

"*Neurodegenerative diseases included some or all of: Huntington's chorea, Parkinson’s disease [PD], amyotrophic lateral sclerosis [ALS], multiple sclerosis [MS], Alzheimer’s disease [AD], frontotemporal dementia [FTD], Wilson’s disease, olivopontocerebellar atrophy [OPCA], progressive supranuclear palsy [PSP], neuroferritinopathy, motor neurone disease (MND), neuromuscular disease and epilepsy."

Literature data sets were fine-tuned using a standard set of inclusion and exclusion criteria. Only original research reports containing numerical data from clinical assessments were included. Non-English articles were excluded, as were reports based on preclinical data or any duplicate reports of previously published data sets.

### Research on the effects of miglustat on dysphagia and outcomes in NP-C

Because NP-C is rare, we conducted broad literature searches to capture any randomised controlled trials comparing miglustat with standard (symptomatic) medical management, as well as evidence from non-randomised studies providing any additional data on miglustat treatment in patients with NP-C.

Initial searches of Medline, Embase, the Cochrane Central Register of Controlled Trials, the National Institutes of Health (NIH) clinical trials database (Clinicaltrials.gov) and the Australian Clinical Trials Registry (Anzctr.org.au) were conducted in January 2010. A further search was then repeated in March 2011 to identify any updated published information. In addition, manual searches were conducted using reference lists from all relevant articles identified in the automated database searches.

## Literature review findings

### Causes of death in NP-C

Out of 741 potentially relevant articles, seven were identified that specifically discussed cause(s) of death in patients with NP-C [8,19,20,37-40]. Additional studies were identified during the literature search, but no data on causes of death were reported. See Additional file 1: Table S1, for a full listing of literature search findings.

Overall, the identified studies included data from 82 patients with NP-C. While there was no specific reference to ‘aspiration pneumonia’ in the identified publications, the most frequent reported cause of death was bronchopneumonia, based mainly on a retrospective case study analysis in 43 UK patients [20] and a chart review of 20 patients in Nova Scotia [19]. While no actual numbers of deaths related to specific causes were reported in the UK-based study, the major cause of death was cited as bronchopulmonary failure with secondary infection [20]. In the Nova Scotia cohort, swallowing difficulties were reported in 100% of patients, and drooling in 95%; pneumonia was cited as the cause of death in 12/20 (60%) patients.

In the wider community, aspiration pneumonia occurs more frequently among individuals who are at risk of aspiration than is reported; in many cases, it goes unrecognised and is not recorded [22]. In the following discussions regarding these and other data, it is important to consider a number of factors that contribute to the under-reporting and/or under-recognition of the role of aspiration pneumonia as a cause of death.

Disease coding systems can complicate the accurate recording of precise causes of death in patients with neurodegenerative diseases. Some coding systems restrict physicians to recording progression of the underlying (primary) disease on death certificates [41,42]. Some physicians also prefer to record a cause of death as ‘pneumonia’ rather than ‘aspiration pneumonia’ [34]. In a large-scale study of dementia patients in England and Wales from 1979 to 2004 [41], only half of deaths that were attributed to ‘AD’ according to the ICD-10 classification would have been coded as such based on ICD-9 criteria, which allowed the specification of bronchopneumonia and dementia. Similar results were reported for PD patients [41].

There is currently a lack of specific or sensitive markers for aspiration, which limits the ability to discern aspiration pneumonia from other forms of pneumonia and results in deaths related to aspiration being recorded under the term ‘pneumonia’ [21,43,44]. Aspiration is considered the predominant mode of infection in nosocomial pneumonia, mostly among the elderly [34]. However, aspiration events are rarely witnessed in patients with aspiration pneumonia, where the clinical presentation typically matches that of community-acquired pneumonia [21]. This is particularly relevant in patients with ‘silent aspiration’, where aspiration occurs without obvious clinical signs of swallowing difficulty [45].

Because of the limited number of precise data on death in NP-C, it may be instructive to refer to data from other, more frequent neurodegenerative diseases that bear similarities to NP-C at the cellular and phenotypic level. For instance, there are a number of common aetiological factors between Huntington's chorea and NP-C at the level of membrane trafficking [46], and patients with these two conditions share certain symptomatologic similarities such as dystonia, motoric difficulties that impair manual and oropharyngeal co-ordination, and impairments to memory and executive functioning that regulate feeding behaviour and the use/retention of safe swallowing strategies. Similarly, AD and NP-C share certain neurodegenerative pathways and neuropathological signs [47,48].

### Causes of death in other neurodegenerative diseases

Data on causes of death in a variety of neurological diseases including Huntington's chorea, amyotrophic lateral sclerosis (ALS), Alzheimer’s disease (AD), frontotemporal dementia (FTD), multiple sclerosis (MS), olivopontocerebellar atrophy (OPCA), Parkinson’s disease (PD), progressive supranuclear palsy (PSP) and Wilson’s disease are listed in Additional file 2: Table S2.

Cause-of-death data were available from 24 out of 1,180 potentially relevant articles. Across all the diseases studied, 20% of patients were classified as having died due to ‘aspiration pneumonia’. Among patients with Huntington's chorea, which probably most resembles NP-C in terms of the profile of motor deficits seen, data from the automated literature search indicated that the overall proportion of deaths due to ‘aspiration pneumonia’ or ‘pneumonia’ was 37.5% (see Additional file 1: Tables S1, Additional file 2: Table S2, Additional file 3: Table S3, Additional file 4: Table S4 and Additional file 5: Table S5). A further two studies were identified during manual searching. In one study, ‘pneumonia’ was the reported cause of death in 55% of patients, among whom 89% died due to ‘aspiration pneumonia’ [44]. In the other study, 42% of deaths were recorded as being due to ‘pneumonia’ [49]. Surprisingly, ‘aspiration pneumonia’ or ‘pneumonia’ were only recorded as causes of death among 11% of ALS patients, despite that fact that most (if not all) ALS patients in the later stages of the disease show some degree of dysphagia [34].

In non-quantitative articles that were excluded from the numerical analysis, aspiration pneumonia was stated as a major cause of death in patients with neurodegenerative disorders, particularly in those with dysphagia due to neurological deterioration [21,22,35]. Precise causes of death were difficult to quantify due to inconsistent reporting, even in studies of more common dementias (where ‘aspiration pneumonia’/’pneumonia’ caused 45% of deaths [50]) and PD (where aspiration pneumonia caused up to 48% of deaths).

### Dysphagia in NP-C and other neurodegenerative diseases

**Figure 1:**
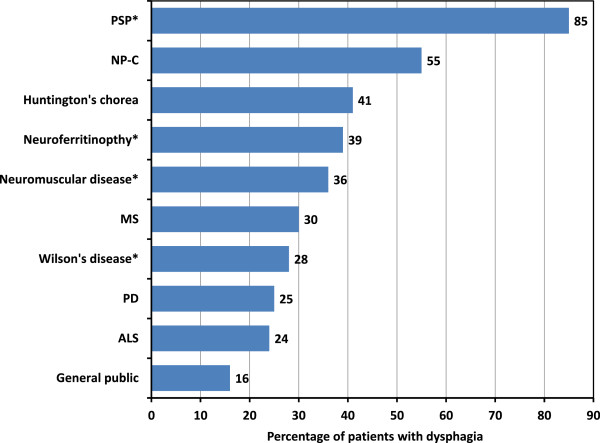
**Prevalence of dysphagia in Niemann-Pick disease type C (NP-C) and other neurodegenerative diseases.** *Conditions where data were available from only one study. ALS, amyotrophic lateral sclerosis; MS, multiple sclerosis, PD, Parkinson’s disease; PSP, progressive supranuclear palsy.

Among 821 potentially relevant articles identified in the search designed to quantify the prevalence of dysphagia in NP-C and other neurodegenerative diseases, data from 52 studies were included for analysis (see Additional file 3: Table S3 for full data listing). Overall, 4,065 from a total of 14,664 patients (28%) had dysphagia. Figure 1 summarises the prevalence of dysphagia in each condition for which quantitative data were available.

The estimated incidence of dysphagia in a large study including PD patients was low at only 8% [51], bringing the unweighted average prevalence for PD down to 25%. However, this study relied on ICD-9 codes for dysphagia. The authors acknowledge that this figure is therefore likely an underestimate. When the results from this study are disregarded, the rate among PD patients becomes 43%, which is closer to other reported findings [52]. Further, the overall incidence for dysphagia among all patients with neurodegenerative diseases becomes 35%.

The incidence of dysphagia reported among ALS patients was surprisingly low (24%). However, the ALS data were strongly influenced by one large study in 3,428 patients that included a range of patients with different ages at onset, and therefore likely comprised a high proportion of patients with early-to mid-stage disease [53]. Dysphagia was reported in 30–50% of patients among other ALS studies, and has been reported in most if not all late-stage ALS patients [34].

Among all neurodegenerative diseases PSP appeared to be associated with the greatest prevalence of dysphagia (85%), although these data were based on only one identified study with evaluable data. Patients with NP-C showed the second-highest prevalence (55%), which is substantially greater than the overall average prevalence among neurodegenerative disease patients. Further, this figure is likely an underestimate, as most patients included in NP-C studies were in the early stages of disease and were being treated with miglustat.

### Association between dysphagia and aspiration pneumonia

Twelve out of 364 potentially relevant articles reported data on the association between dysphagia and aspiration pneumonia in patients with neurodegenerative disease, stroke or traumatic brain injury (see Additional file 4 for full data listing). Based on relative risk analyses, these published data consistently indicated an increased risk of aspiration pneumonia where dysphagia was present (Table 1). Relative risk values ranged from 1.6 (95% CI 0.1, 38.0) to 126 (95% CI 8, 2065).

**Table 1:** Relative risk of aspiration pneumonia in patients with neurodegenerative disease or stroke and dysphagia

**Source, author (year)**	**Patient type**	Relative risk*
		**(95% CI)**
Ahn et al. (2010) [54]	Stroke	106 (6, 1729)
Alshekhlee et al. (2010) [55]	Stroke	30 (4, 218)
Altman et al. (2010) [56]	Dysphagia patients	13 (12, 13)
Aviv et al. (1997) [57]	Stroke	13 (1, 216)
Chua and Kong (1996) [58]	Stroke	4.6 (1.0, 20.5)
Daniels et al. (1998) [36]	Stroke	1.6 (0.1, 38.0)
James (1998) [59]	Stroke	4.1 (1.5, 11.4)
Meng et al. (2000) [60]	Stroke	2.4 (0.1, 40.1)
Perry and McLaren (2000) [61]	Stroke	21.0 (2.8, 155.9)
Schurr et al. (1999) [62]	Traumatic brain injury	2.9 (0.1, 67.3)
Spencer et al. (2009) [63]	Stroke	11.0 (1.0, 178.3)
Sung et al. (2010) [64]	Parkinson’s disease	126 (8, 2065)

*Relative risk calculated using Mantel-Haenszel fixed effects method.

**Figure 2:**
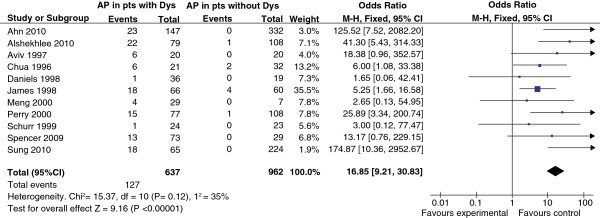
**Odds ratios for association between dysphagia and aspiration pneumonia in patients with neurodegenerative diseases and stroke.** M-H, Mantel-Haenszel fixed effects method. Note: Altman et al. [56] paper excluded.

Data from this literature search were combined for meta-analysis (Figure 2). After exclusion of one very large study [56], which included data from 77,540,204 general hospital patients resulting in unacceptably high weighting of the overall sample (accounting for 99.8% of all included patients), odds ratios indicated a highly significantly increased risk of aspiration pneumonia among patients with dysphagia. The overall odds ratio for developing pneumonia was 16.85 (95% CI 9.21, 30.83), with values ranging from 1.7 (95% CI 0.1, 42.4) to 174 (95% CI 10, 2,952). Analysis including data from the large general hospital population study confirmed this finding (odds ratio, 14.1 (95% CI 13.9, 14.3))

### Association between aspiration pneumonia and mortality

Five of 243 potentially relevant articles were identified that reported data on both aspiration pneumonia and mortality outcomes in patients with PD, stroke or epilepsy (see Additional file 5: Table S5 for full data listing). One extra article was identified in an additional hand search of the literature that contained data from a mixed population of patients with PD, MND, AD or Huntington's chorea [33]. No published articles were identified that reported data on both aspiration pneumonia and mortality in patients with NP-C.

**Figure 3:**
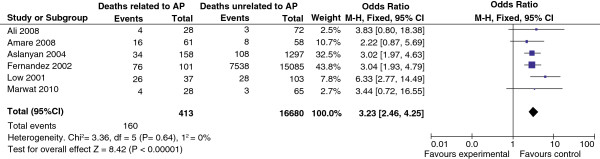
**Odds ratios for association between aspiration pneumonia and mortality outcomes.** M-H, Mantel-Haenszel fixed effects method.

The relative risk (95% CI) of mortality among patients with aspiration pneumonia in the identified studies ranged from 1.51 (1.35,1.69) to 3.42 (0.82, 14.35) (Table 2). When data were combined in a meta-analysis to evaluate the relationship between these two outcomes, patients experiencing aspiration pneumonia were seen to have an increased risk of mortality (Figure 3). The overall odds ratio for mortality was 3.23 (95% CI 2.46, 4.25).

**Table 2:** Relative risk of mortality in patients with neurodegenerative disease or stroke and aspiration pneumonia

**Source, author (year)**	**Patient type**	Relative risk*
		**(95% CI)**
Ali et al. (2008) [65]	Stroke	3.42 (0.82, 14.35)
Amare and Amanuel (2008) [66]	Epilepsy	1.90 (0.88, 4.10)
Aslanyan et al. (2004) [67]	Stroke	2.58 (1.82, 3.66)
Fernandez and Lapane (2002) [68]	Parkinson’s disease	1.51 (1.35,1.69)
Low et al. (2001) [33]	Dysphagia patients	2.58 (1.77,3.78)
Marwat et al. (2010) [69]	Stroke	3.10 (0.74, 12.93)

*Relative risk calculated using Mantel-Haenszel fixed effects method.

## Epidemiological research: overall findings

The quantitative analysis of causes of death in NP-C patients is currently limited by the scarcity of relevant published studies, with few reports containing mortality data. There was limited but consistent reporting of bronchopneumonia as the most common cause of death in NP-C, accounting for over 60% of patients [19,20]. Supplemental analysis of data in other neurodegenerative diseases that feature dysphagia showed ‘aspiration pneumonia’ or ‘pneumonia’ reported as a cause of death in over 20% of patients. However, this is considered to be vastly underestimated due to a number of factors that contribute to under-reporting of aspiration pneumonia as cause of death.

The analysis of published data in NP-C and a range of other neurodegenerative diseases showed that dysphagia is a frequent symptom, particularly in NP-C and PSP. Further, relative risk data and meta-analyses provide clear evidence that patients with dysphagia are at an increased risk of developing aspiration pneumonia, and that there is a strong link between the occurrence of aspiration pneumonia and mortality risk. With these links now established, the following sections specifically examine the effects of miglustat therapy on dysphagia and mortality risk in NP-C.

## Studies and methods assessing the effects of miglustat on dysphagia in NP-C

All published clinical studies providing data on swallowing function in miglustat-treated patients are summarised in Table 3.

**Table 3:** Summary of randomised and non-randomised studies with information on the effects of miglustat on dysphagia

**Trial ID [reference]**	**Design**	**Treatment**	**Patients**	**Swallowing function**	**Key findings**
OGT-918-007 [25]	12-month randomised, controlled Phase II study comparing miglustat with standard (symptomatic) therapy	Main study: miglustat 200 mg t.i.d. (n = 20) vs. standard care (n = 9)	Main study: male and female adults and juveniles (aged ≥12 years)	Ability to swallow different foods (5 mL of water, 1 teaspoon of puree, 1 teaspoon of soft lumps, or a third of a cookie)	Improved ability to swallow water in 6 patients (30%), puree in 3 patients (15%), soft lumps in 3 patients (15%), and a third of a cookie in 7 patients (35%) after 12 months of miglustat therapy
		Sub-study: miglustat 200 mg t.i.d adjusted for BSA (n = 12)	Sub study: male and female children aged 4–11 years	Assessed at 6 and 12 months or withdrawal/follow-up	Over 80% of children had normal swallowing at baseline
OGT-918-007 ext (a) [27]	Prospective, non-controlled, 12-month extension to OGT-918-007	Miglustat 200 mg t.i.d.	Male and female adults and juveniles (aged ≥12 years) who received miglustat (n = 17) or standard care (n = 8) for 12 months	Swallowing assessment (as above) at 12 and 24 months and last visit	Swallowing improved/stable (*vs.* baseline) in 86% of patients completing 12 months, and 79–93% of those completing 24 months on miglustat
OGT-918-007 ext (b) [26]	Prospective, non-controlled, 12-month extension to OGT-918-007 sub-study	Miglustat 200 mg t.i.d adjusted for BSA	Male and female children aged 4–11 years who underwent 12 months of miglustat therapy (n = 10)	Swallowing assessment (as above) at 12 and 24 months and last visit	Nine patients (90%) had normal swallowing function at both baseline and Month 24
NP-C retrospective Stage 1 survey [70]	Retrospective, multicentre observational cohort study	Adults ≥18 years (n = 14): miglustat 200 mg t.i.d.	Patients previously or currently treated with miglustat in clinical practice settings	Dysphagia subscale of NP-C disability scale [12]	Continuous deterioration prior to initiation of miglustat therapy
		Juveniles 12–17 years (n = 13): miglustat 200 mg t.i.d.			Similar proportions of patients in each swallowing disability category at treatment start and last post-treatment assessment (stabilisation)
		Paediatrics ≤12 years (n = 34): miglustat adjusted for BSA			
Spanish/Portuguese paediatric cohort study [11]	Multicentre observational chart review	Miglustat 200 mg t.i.d. adjusted for BSA in symptomatic patients (n = 16)	Male and female paediatric patients treated in Spain and Portugal	Dysphagia subscale of a modified NP-C disability scale [11]	Stable neurological manifestations (including swallowing) in juvenile-onset patients
		Symptomatic therapy in 1 asymptomatic patient			Smaller therapeutic benefits in younger-onset patients with greater disease severity at baseline
Italian case series [18]	Longitudinal case series of Italian patients	Miglustat 250–300 mg/mq/day in three divided doses for up to 4 years	Male and female patients treated for ≥3 years, with swallowing function assessed by VFSS (n = 4)	VFSS	Improved swallowing in patients with dysphagia/aspiration at baseline (n = 3)
					No deterioration in the patient with normal swallowing at baseline
Taiwanese data [29]	Longitudinal case reports	Miglustat 200 mg t.i.d. adjusted for BSA for 1 year	Young male patients, 1 with severe swallowing impairment and 1 with impaired language/speech, who underwent serial VFSS	VFSS	Patient 1: substantially improved swallowing function after 6 months
					Patient 2: normal swallowing before and throughout therapy

BSA = body surface area; VFSS = videofluoroscopic studies.

### Swallowing assessments based on clinical judgment

Swallowing function data were available from a 12-month randomised, controlled trial that assessed the efficacy, safety and tolerability of miglustat 200 mg t.i.d. in juvenile and adult patients aged ≥12 years (n = 29), compared with standard care [25]. Findings were also reported from a parallel non-controlled sub-study of this trial, which assessed miglustat in children aged 4–11 years (n = 12) [25].

Long-term data from patients continuing miglustat therapy during 12-month open-label extensions of the main juvenile/adult study (n = 21) and the paediatric sub-study (n = 10) have since been published [26,27]. In each of these reports dysphagia was assessed based on standardised clinical assessments of the patients’ ability to swallow different foods (e.g. water, puree, soft pasta/noodles, a cookie), graded using a five-point categorical scale: ‘no problems swallowing’, ‘mild problems’, ‘moderate problems’, ‘severe problems’, or ‘could not swallow at all’.

Retrospective data on swallowing function were reported in an international, multicentre, observational cohort study that evaluated neurological disease progression in patients treated with miglustat in clinical practice settings (n = 66) [70]. Swallowing function was evaluated based on patients’ scores on the dysphagia subscale of a modified, disease-specific disability scale [12]. Using this subscale the degree of dysphagia before miglustat therapy, at treatment initiation, and after therapy was rated as ‘normal’ (score = 0), ‘occasional dysphagia’ (score = 0.33), ‘daily dysphagia’ (score = 0.66), or ‘nasogastric tube or gastric button feeding’ (score = 1) [70]. Data then underwent categorical analysis for ‘improvement’, ‘stabilisation’ or ‘worsening’ of swallowing function.

### Swallowing assessments based on instrumental methods

Findings from instrumental, quantitative assessments of swallowing have been reported from two studies [18,29]. Both reports were based on longitudinal case analyses incorporating videofluoroscopic studies (VFSS), which are considered the gold standard method for studying oropharyngeal swallowing function. VFSS allow the in-depth evaluation of all phases of the swallowing motion, enabling clear distinctions between oral-phase and pharyngeal phase dysfunction [71].

Fecarotta et al. reported findings from detailed serial studies of swallowing function in three female patients with late-infantile or juvenile-onset NPC1 and one male patient with severe early-infantile onset NPC2 [18]. These patients received miglustat therapy, dosed according to BSA for between 3 and 4 years. The severity of dysphagia was evaluated at 2–6 monthly intervals based on an adapted version of the 6-point Dysphagia Severity Score (DSS) [71], which ranges from normal swallowing (score = 0) to severe dysphagia with potential for aspiration (score = 5). A 7-point Penetration-Aspiration Score (PAS) was also applied at each VFSS assessment to quantify penetration/aspiration in the airways according to published methods [71]; again, higher scores on this scale indicated more severe impairment. VFSS were also conducted in parallel with NP-C disability scale assessments to assess correlations between swallowing function, overall neurological deterioration (based on composite disability scores [70]) and the dysphagia subscale scores.

Chien et al. reported data from two young symptomatic male patients with juvenile-onset NP-C who completed 1 year of miglustat therapy, dosed according to body surface area. One patient had severely impaired swallowing function, and the second displayed normal swallowing but impaired language and speaking ability prior to miglustat therapy [29]. Videotapes of VFSS of liquid barium swallowing were analysed by a single radiologist based on the Han functional dysphagia scale [72]. Scores were assigned to 11 variables that covered the ‘entire swallowing cycle’ or ‘all aspects of the oropharyngeal swallow’. Individual scores assessing each of these variables were summated to provide a total functional dysphagia score ranging from 0 (no impairment) to 100 (severe impairment).

## Results from studies of the effects of miglustat on dysphagia in NP-C

### Categorical data findings

Based on clinical judgment, miglustat was reported to stabilise or improve swallowing function in a substantial proportion of patients with NP-C.

**Figure 4:**
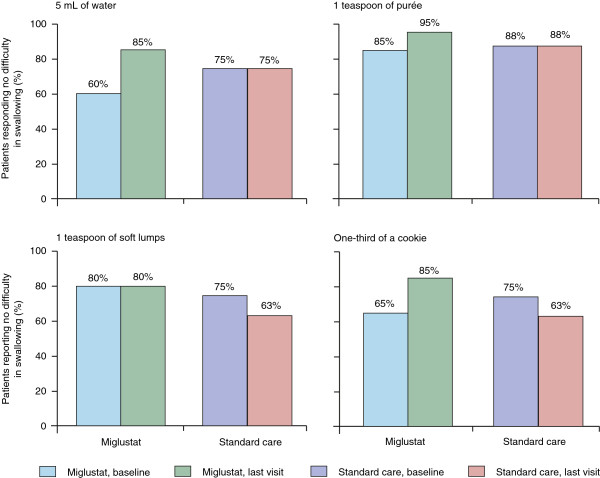
**Effect of miglustat *versus *standard care on swallowing ability in adolescent/adult patients over 12 months in a randomised, controlled trial.** Reproduced with permission from [25].

In the randomised controlled trial, improvements were seen in the ability to swallow water for six patients (30%), puree for three patients (15%), soft lumps for three patients (15%), and a third of a cookie for seven patients (35%) after 12 months of therapy [25]. The proportions of miglustat-treated juvenile/adult patients reporting no difficulty swallowing water, puree and one-third of a cookie after 12 months of miglustat therapy increased from baseline by 10–25% (Figure 4). In contrast, the proportions of juvenile/adult patients on standard care who showed no difficulty swallowing were either equal to or lower than baseline at 12 months.

In the non-controlled extension study [27], swallowing was improved or stable (*versus* baseline) in 86% of all juvenile/adult patients who completed 12 months of miglustat therapy (n = 21), and in 79–93% of those completing 24 months on miglustat (n = 15), depending on the substance assessed.

Swallowing difficulties were less common in the paediatric sub-study population (recorded in only 33% of patients at baseline) [25]. Over 80% of children swallowed all four test substances easily before treatment was started. It was therefore anticipated that improvements in swallowing were not likely. A worsening in the ability to swallow water, puree, soft lumps and one-third of a cookie was noted after 12 months of therapy in three patients (27%), two patients (18%), one patient (9%) and two patients (18%), respectively. However, among 10 paediatric patients who participated in and completed extension treatment, only one showed deterioration in the ability to swallow one-third of a cookie after 24 months of therapy. All other patients showed no change in swallowing function from baseline.

Dysphagia assessments in NP-C patients included in the retrospective observational NP-C cohort indicated similar findings among patients treated outside the context of clinical trials [70]. The retrospective cohort comprised 66 patients with a mean (SD) age at diagnosis of 9.7 (7.6) years; the mean (SD) age at treatment start was 12.8 (9.5) years. On average, patients had been under observation for a mean (SD) period of 3.1 (3.4) years between diagnosis and initiation of miglustat therapy.

In line with other data on the natural history of NP-C [10,16], composite NP-C disability scale scores indicated marked neurological deterioration before the initiation of miglustat therapy [70]. On the dysphagia subscale, while 95% of patients had normal swallowing function or occasional dysphagia at diagnosis, this had dropped to 66% by the end of the pre-treatment observation period. In contrast, the proportion of patients with normal swallowing or occasional dysphagia after a median (range) of 1.5 (0.1–4.5) years’ miglustat treatment was almost identical to that at the start of therapy (67%). Overall, 51/63 (81.0%) patients showed a stable/improved score on the dysphagia subscale during treatment.

### Instrumental assessments of swallowing

Findings from direct instrumental assessments of the effects of miglustat on swallowing function, based on VFSS [18,29], support categorical data based on clinical observation of dysphagia [25-27,70].

In the Italian case series, where serial VFSS were conducted to quantify changes in swallowing function during 36–48 months of miglustat therapy, all three patients with dysphagia at treatment start showed early improvements in swallowing ability (DSS scores), and one patient who did not exhibit dysphagia before therapy showed stable swallowing function throughout treatment [18]. Improvements in swallowing ability, particularly in terms of pharyngeal phase function, occurred in parallel with improvements or stabilisation of overall neurological manifestations assessed by composite NP-C disability scores. These apparent long-term beneficial effects of miglustat on swallowing function were associated with sustained reductions in penetration/aspiration (based on PAS scores) in all patients who showed severe swallowing impairment and aspiration prior to treatment.

Impairments of pharyngeal swallowing function tended to occur later in the course of NP-C than oral-phase impairment, and more severe pharyngeal-phase involvement associated with penetration/aspiration of contrast agent in the airways was present in patients with the most severe overall neurological impairment [18]. Further, in all patients in this case series, improvements in pharyngeal swallowing function during miglustat therapy were greater, and occurred earlier, than those in the oral phase. Modest impairment in the preparatory/oral phase persisted during follow up in two patients with severe swallowing impairment (and higher NP-C disability scale scores) at treatment start.

It can be speculated that the apparent difference in the effects of miglustat on pharyngeal *versus* oral-phase swallowing function might reflect selective therapeutic effects of miglustat on the neurological pathways that control them [18]. While the preparatory/oral phase is activated by peripheral receptors as well as through stimulation of sensory cortical neurones, the pharyngeal and oesophageal phases are mediated by involuntary reflexes dependent on brainstem neurones. Since greater therapeutic effects were noted for pharyngeal phase swallowing, it might be that miglustat affects autonomic, brainstem-based neuronal circuits before higher, cortical centres in NP-C. The significant effects of miglustat on saccadic eye movements (which are also modulated by pathways in the brainstem) during the randomised clinical trial would certainly seem to support this [25].

Data reported from two male Taiwanese patients support findings from the Italian cohort [29]. In the patient with pronounced swallowing impairment, who had displayed neurological manifestations since 5 years of age, VFSS identified severe oropharyngeal dysphagia and prominent aspiration before miglustat treatment was initiated at the age of 14 years. Distinct improvements in swallowing function were seen after 6 months of therapy, reflected by a 25% reduction in Han functional dysphagia scale score that was sustained through to Month 12. Similar to findings reported in the Italian case series [18], this patient’s improved swallowing function occurred simultaneously with improvements in ambulation [29]. The second patient, who had displayed splenomegaly since birth and behavioural problems (Asperger-like syndrome) since the age of 8 years, did not display significant swallowing impairment either before treatment or during therapy, but did show improvements in communication, social interaction and cognitive function by Month 12 [29].

### Data limitations

In assessing the available published data on the effects of miglustat on dysphagia in NP-C, a number of limitations should be taken into account. NP-C is markedly heterogeneous and different patient cohorts have limited comparability, particularly in terms of age at neurological disease onset [1,10]. Most published evidence related to changes in swallowing function during miglustat therapy stems from subjective assessments (i.e. clinical observation and disability scales) rather than direct, quantitative swallowing studies such as those employed in the Italian cohort and Taiwanese case reports [18,29]. Dysphagia in NP-C often has mixed motor and sensory components, and silent aspiration of small or trace amounts of food or fluid is not well diagnosed without direct VFSS. Finally, the inclusion in this analysis of the retrospective miglustat observational cohort study [70] introduces potential bias, so the contribution of these data, albeit based on an established disease-specific disability scale, should be considered with caution.

## Longitudinal analysis of survival in miglustat-treated patients

### Analysis methods

To identify the effect of miglustat on patient survival, longitudinal statistical analyses were conducted based on all available published data from miglustat-treated patients and from an untreated cohort. Patient age at onset of neurological manifestations has previously been shown to have a strong influence on the severity, progression and prognosis of NP-C [1,10,15,70]. An analysis of patient survival was therefore conducted based on subgroups of patients, categorised by age at neurological onset, from the overall groups of untreated and treated patients identified during our analyses of published data. Patient survival was then compared between the overall treated and untreated patient groups.

Survival analyses were based on both univariate and multivariate methods. A univariate Kaplan-Meier analysis using the log-rank test evaluated survival over time, and multivariate Cox proportional hazards modelling was performed to provide estimates of overall mortality risk per treatment group and according to patient age.

Analyses in miglustat-treated patients were based on time from the start of therapy as ‘age at neurological disease onset’ data were not available for many patients. However, analyses in untreated patients were based on time from onset of neurological manifestations, as most patients had available data [1].

### Results

The untreated group comprised patients from the French NP-C cohort [1]. This population provided longitudinal data from a total of 97 patients diagnosed in French hospitals (Table 4). Nineteen patients were excluded from this control cohort as they died during the first days or months of life, and would therefore not have been eligible to receive miglustat therapy (see *Data limitations*).

**Table 4:** Mortality in untreated and miglustat-treated NP-C patients included in survival analysis by age at neurological disease onset

	**Death, n (%)**			
		**Untreated**		**Treated**
	**n**	**(N = 97)**	**n**	**(N = 90)**
**Overall**		74 (76)		3 (3)
Age category*				
<2 years	27	27 (100)	4	0 (0)
2–11 years	48	40 (83)	37	2 (5)
>11 years	22	7 (32)	49	1 (2)

*Age at neurological symptom onset in untreated patients, and age at treatment start in treated patients.

The treated group (N = 90) comprised patients from the randomised controlled trial (n = 25) [25], the Italian cohort (n = 4) [18], and the retrospective observational cohort (n = 61) (Table 4). A number of patients were excluded from this data set due to non-availability of data, or to avoid overlap between different publications based on the same patients. Seven patients from the randomised trial data set were excluded. Six of these cases did not have a time of neurological onset recorded at baseline, disallowing their inclusion in the analysis. The seventh patient only received miglustat for 71 days, which made it very unlikely that a visible therapeutic response could be expected. Taiwanese patients (n = 2) [29] and patients from a Spanish clinical experience cohort (n = 16) [11] were excluded as they formed part of the retrospective cohort.

**Figure 5:**
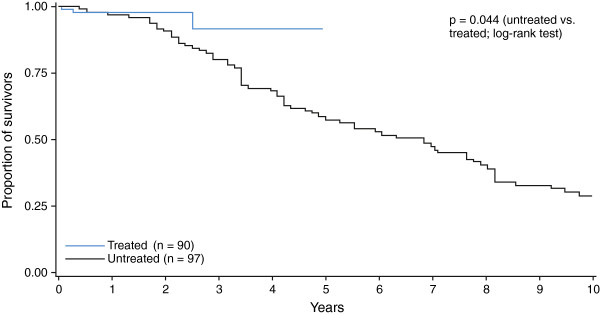
**Influence of age at neurological onset on patient survival in untreated NP-C patients.** Kaplan-Meier log-rank analysis of mortality by age at neurological disease onset in untreated patients.

Findings from Kaplan-Meier log-rank analysis in different age-at-onset subgroups of untreated patients were in line with previous data on the natural history of NP-C [10]. Untreated patients with neurological disease onset at <2 years of age showed the lowest survival rate over a 10-year period of follow up, while patients with onset aged 11+ years had the best survival rate (Figure 5).

Analyses of survival by age at neurological onset among miglustat-treated patients were limited by low patient numbers and relative lack of long-term follow up among the available published data. No Kaplan-Meier curve was possible for treated patients with disease onset at <2 years of age as there were no deaths in this subgroup. Likewise, no meaningful analysis was possible for treated patients with neurological onset at >11 years of age, as only one patient in this subgroup died (early during miglustat therapy). Kaplan-Meier analysis was only possible for the 2–11 year age at onset subgroup, and showed a numerically better survival rate (2 deaths among 37 patients) during approximately 2.5 years of follow up *versus* the equivalent age subgroup of untreated patients (40 deaths among 48 patients).

**Figure 6:**
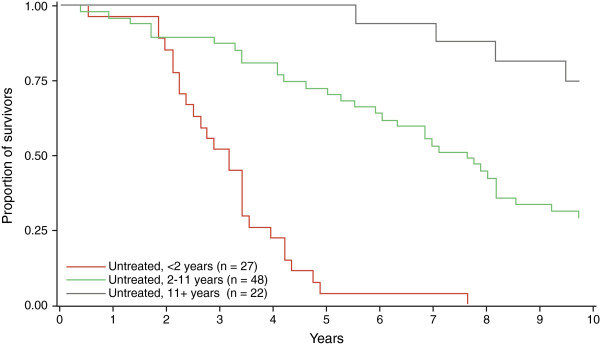
**Influence of miglustat therapy on NP-C patient survival.** Kaplan-Meier log-rank analysis of mortality since treatment start for treated patients and since age at neurological disease onset in untreated patients.

In the overall treated *versus* untreated group analysis, a total of 74 patients died in the untreated group compared with just three patients in the treated group, leading to a large numerical difference in the overall mortality rates between the two groups; 76% *versus* 3%, respectively. However, Cox proportional hazard modelling did not establish statistical significance for this difference after adjusting for age (p = 0.34; hazard ratio [95% CI] for treated vs. untreated, 0.56; 0.17, 1.86), likely due to the very low number of deaths and relatively short follow-up time in the treated group. Kaplan-Meier analysis with univariate log-rank testing identified a significant difference in mortality between the treated and untreated groups (p = 0.044) (Figure 6).

Patient age at treatment start (in treated patients) and age at neurological symptom onset (in untreated patients) were found to have a very significant influence on survival. Cox proportional hazard modelling showed that, among all patients (treated and untreated), patients aged <2 years had a significantly higher mortality rate (87%, p < 0.0001; hazard ratio [95% CI], 5.56 [3.08, 10.13]) compared with 49% in patients aged 2–10 years (the reference group). Conversely, patients aged >11 years had a significantly lower rate (11%, p = 0.0003; hazard ratio [95% CI], 11% [0.10, 0.51]) compared with the reference group.

### Data limitations

A number of data limitations should be acknowledged in considering the results of these survival analyses, which are chiefly related to the composition and nature of the treated cohort compared with the untreated cohort. The untreated patient data used in these statistical comparisons are derived solely from the French NP-C cohort, while data from miglustat-treated patients are derived from a multinational group of patients who were included in a number of different studies.

There were large differences in the length of follow up in treated patients compared with untreated patients. Follow-up data were available from ap-proximately 1 month to 5 years among patients included in the randomised controlled trial, retrospective cohort study and Italian cohort [25,70,73], compared with approximately 23 years in the untreated cohort [1]. More specifically, while the unavailability of data for age at onset of neurological manifestations for many of the treated patients necessitated a comparison of survival from treatment start (in treated patients) with survival from neurological disease onset (in untreated patients), such a comparison presents difficulties.

Published data indicate that substantial periods of time (and likely, significant disease progression) may pass between neurological onset and initiation of miglustat treatment, due either to diagnostic delay or the date at which miglustat became available (or both) [11,70]. Thus, some patients in the treated group might have progressed significantly before treatment initiation, while in some treated patients miglustat therapy might have been initiated after a relatively short period of time after neurological disease onset. However, given the low numbers of patients available for this assessment, it is not possible to properly assess the influence of this difference.

The untreated patient data constitute an unmatched (and generally younger) control cohort compared with the treated patients in terms of age. The mean (SD) age at treatment start in the randomised controlled trial of miglustat was 25.4 (9.8) years in adolescent/adult patients (range 12–42 years; n = 20) and 7.2 (2.5) years in paediatric patients (range 4–11 years; n = 12) [25]. The mean (SD) age at treatment start in the retrospective NP-C cohort study was 12.8 (9.5) years (range 0.6–43 years; n = 66). The mean age at treatment start in the Italian case series was 8.0 (4.9) years (range 0.9–12 years; n = 4). In contrast, the patient age at neurological symptom onset ranged between approximately 6 months and 55 years; 50% of patients were aged <5 years at onset, and a further 25% were aged <10 years. While both the Kaplan-Meier and Cox proportional hazard modelling analyses of patient survival were controlled for patient age, it cannot be discounted that age-related disease natural history might still have had an influence on the apparent difference in patient survival between the treated and non-treated groups.

It is possible that differences in regional population characteristics (e.g. lifestyle, diet, symptomatic therapies) between the treated and untreated cohorts could have contributed to, or in some other way influenced, the apparent treatment difference. However, it should be remembered that NP-C is pan-ethnic and occurs sporadically across populations, regardless of race. Differences in race *per se* were not expected to affect the study results.

Finally, while each of the published studies assessing miglustat therapy that were identified in this analysis made efforts to minimise bias, a number of sources of potential bias were inherent following their inclusion in the overall treated group. In particular, while there was minimal selection bias in the randomised, controlled trial of miglustat in NP-C, neither severely affected patients nor symptom-free patients were included in the study [25]. The exclusion of severely affected patients may remove those who were unlikely to improve, resulting in a positive bias (i.e. inclusion of patients more likely to improve). Conversely, non-inclusion of asymptomatic patients might exclude those whose symptom onset may be significantly delayed, thus extending the apparent survival (negative bias).

## Safety and tolerability of miglustat in NP-C

While miglustat has been shown to be generally well tolerated in patients with NP-C, the perceived safety and tolerability profile of any drug can affect treatment compliance and can thus impact on clinical efficacy. Similar to previous observations in patients with Gaucher disease type 1 [23,74-78], the principal adverse events observed during clinical studies with miglustat in patients NP-C were gastrointestinal in nature [11,23,25-27,79].

During clinical trials and subsequent clinical experience, diarrhoea, flatulence, bloating and abdominal pain/discomfort were the most commonly reported adverse events associated with miglustat, particularly during the initial weeks/months of therapy [79]. While frequently observed, these gastrointestinal disturbances are generally mild or moderate in severity and usually resolve spontaneously with time on continued therapy [79]. Where required, diarrhoea can effectively be controlled using anti-propulsive medications, used according to the respective manufacturers’ prescribing information. Dietary modifications such as restriction of disaccharides or general, controlled reductions in overall carbohydrate intake can improve the gastrointestinal tolerability of miglustat, particularly if initiated before the start of therapy. Such dietary modifications should be undertaken over a course of weeks or months, with patients gradually being re-introduced to a normal diet dependent on tolerability [79,80].

## Conclusions

Published data indicate that aspiration pneumonia is the most common cause of death in neurodegenerative diseases, including NP-C, and that dysphagia can reliably be considered as a risk factor for mortality as it is a frequent cause of aspiration pneumonia.

Miglustat has been shown to stabilise neurological manifestations of NP-C. Beneficial effects of miglustat on swallowing function have been reported in a number of previously published studies based on clinical judgment and quantitative, instrumental VFSS.

Our findings based on systematic literature analyses and statistical survival evaluations suggest that NP-C patients receiving miglustat therapy may have a greater lifespan compared with untreated patients, and that this effect is likely related to a beneficial effect of miglustat on dysphagia. Because of the extremely variable nature of this disease and the limited published data, further, longer-term data are required to confirm this apparent effect.

## Abbreviations

AD: Alzheimer’s disease; ALS: Amyotrophic lateral sclerosis; BSA: Body surface area; CI: Confidence interval; DSS: Dysphagia severity score; FTD: Frontotemporal dementia; HD: Huntington’s disease; ICD 9/10: International Classification of Diseases versions 9 and 10; MND: Motor neurone disease; MS: Multiple sclerosis; NP-C: Niemann-Pick disease type C; *NPC1/NPC2*: Specific gene mutations in patients with NP-C; OPCA: Olivopontocerebellar atrophy; PAS: Penetration-Aspiration Score; PD: Parkinson’s disease; PSP: Progressive supranuclear palsy; SD: Standard deviation; SEM: Saccadic eye movement; VFSS: Videofluoroscopic studies.

## Competing interests

None of the authors received honoraria for their roles in the production of this article. MW has received travel expenses, research grant funds and consulting honoraria from Actelion Pharmaceuticals Ltd. Y-HC has received consultancy honoraria from Actelion Pharmaceuticals Ltd. JI has received travel expenses and consultancy honoraria from Actelion Pharmaceuticals Ltd. DR is an employee of Actelion Pharmaceuticals pty Ltd, Australia. DS has received an educational grant from Actelion Pharmaceuticals pty Ltd. MCP has received a research grant, travel expenses, and consulting honoraria (directed to Mayo Clinic) from Actelion Pharmaceuticals Ltd; travel expenses and consulting honoraria (directed to the Mayo Clinic) from Shire Human Genetic Therapies, an honorarium for acting as the Chair of a Data Monitoring Committee from Stem Cells Inc., and royalties as an editor of *Up-To-Date*. He receives research funding from the National Institutes of Health, and has received travel expenses from the Institute of Medicine for service on the Committee to Review Adverse Effects of Vaccines.

## Authors' contributions

All authors have reviewed and interpreted the data, reviewed each draft of the manuscript and approved the final version for submission. All authors read and approved the final manuscript.

## Supplementary Material

Additional file 1 **Table S1.** Results for literature review examining cause of death in patients with NP-C. Click here for file

Additional file 2 **Table S2.** Literature search results for the cause of death in neurodegenerative diseases [81-102]. Click here for file

Additional file 3 **Table S3.** Results for literature search to determine the prevalence of dysphagia in neurodegenerative diseases including NP-C [103-142]. Click here for file

Additional file 4 **Table S4.**Literature search results for the association between dysphagia and aspiration pneumonia. Click here for file

Additional file 5 **Table S5.** Literature search results regarding the association between aspiration pneumonia and mortality. Click here for file
