# Supplementary material for: Dysphagia as a risk factor for mortality in Niemann-Pick disease type C: systematic literature review and evidence from studies with miglustat
Source: Orphanet J Rare Dis. 2012 Oct 6;7:76. doi: 10.1186/1750-1172-7-76 (PMC3552828; doi:10.1186/1750-1172-7-76)
Supplement: Additional file 1 — Table S1. Results for literature review examining cause of death in patients with NP-C. [file 1750-1172-7-76-S1.doc]

**Table S1. Results for literature review examining cause of death in patients with NP-C**

| **Author / country** | **Study design** | **N** | **No. deaths** | **Cause of death** |
| --- | --- | --- | --- | --- |
| Bjurulf et al. [37]  Norway | Case study | 2 | 2 | Respiratory failure (n = 2) |
| Harzer et al. [38]  Germany | Case study | 2 | 2 | Fever (n = 1), pregnancy (n = 1) |
| Jan et al. [19]  Canada | Cohort chart review | 20 | 20 | Pneumonia (n = 12; 61%) |
| Kawai et al. [39]  Japan | Case report | 1 | 1 | Stridor and dyspnoea |
| Kelly et al. [20]  UK | Retrospective case series | 43 | 28 | Bronchopulmonary failure with secondary infection |
| Landas et al. [40]  USA | Case study | 1 | 1 | Coronary artery disease |
| Sevin et al. [8]  France | Case series | 13 | 5 | Septicaemia (n = 1), post-traumatic cerebral haemorrhage (n = 1), no cause specified (n = 3) |
