# Supplementary material for: Dysphagia as a risk factor for mortality in Niemann-Pick disease type C: systematic literature review and evidence from studies with miglustat
Source: Orphanet J Rare Dis. 2012 Oct 6;7:76. doi: 10.1186/1750-1172-7-76 (PMC3552828; doi:10.1186/1750-1172-7-76)
Supplement: Additional file 2 — Table S2. Literature search results for the cause of death in neurodegenerative diseases [81-102]. [file 1750-1172-7-76-S2.doc]

**Table S2. Literature search results for the cause of death in neurodegenerative diseases**

| **Author / country** | **Study design** | **N** | **No. deaths** | **No. deaths due to AP/ pneumonia** | **No. deaths due to other causes** | **No. deaths due to unspecified causes** |
| --- | --- | --- | --- | --- | --- | --- |
| **ALS** |  |  |  |  |  |  |
| Chio et al. [83]  Italy | Prospective registry search | 134 | 114 | 0 | 108 | 6 |
| Gil et al. [84]  France | Prospective medical chart review | 302 | 302 | 42 | 221 | 39 |
| Lewis et al. [85]  UK | Contemporaneous case study analysis | 104 | 7 | 0 | 7 | 0 |
| Lo Coco [86]  Italy | Prospective case study analysis | 33 | 3 | 3 | 0 | 0 |
| Total number (%) |  |  | 426 | 45 (10.6) | 336 (78.9) | 45 (10.6) |
| **Dementia** |  |  |  |  |  |  |
| Brunnstrom et al. [50]  Sweden | Retrospective autopsy review | 524 | 524 | 236 | 254 | 34 |
| Total number (%) |  |  | 524 | 236 (45.0) | 254 (48.5) | 34 (6.5) |
| **Huntington's chorea** |  |  |  |  |  |  |
| Lipe et al. [87]  USA | Retrospective observational study | 34 | 7 | 2 | 5 | 0 |
| Wojaczybska-Stanek  Poland | Case study | 1 | 1 | 1 | 0 | 0 |
| Sorenson et al. [49]  International | Retrospective death certificate survey | 395 | 395 | 166 | 229 | 79 |
| Heemskerk et al. [44]  International | Literature survey | 224 | 224 | 124 | – | – |
| Total number (%) |  |  | 627 | 293 (46.7) | 234 (37.3) | 79 (12.6) |
| **MS** |  |  |  |  |  |  |
| Deshpande et al. [88]  Canada | Natural history cohort study | 1043 | 312 | 102 | 210 | 0 |
| Ebers et al. [89]  UK | Double-blind, placebo-controlled study | 372 | 8 | 1 | 7 | 0 |
| Farge [90]  France | Retrospective observational study | 345 | 35 | 2 | 32 | 1 |
| Smestad [91]  Norway | Retrospective cohort | 225 | 225 | 4 | 212 | 9 |
| Sumelahti et al. [92]  Finland | Longitudinal cohort | 32165 | 464 | 16 | 448 | 0 |
| Total number (%) |  |  | 1044 | 125 (12.0) | 909 (87.1) | 10 (1.0) |
| Ben-Shlomo [93]* | Prospective | 220 | 99 | 0 | 99 | 0 |
| Beyer et al. [93]*  Italy | Prospective | 84 | 50 | 17 | 33 | 0 |
| D'Amelio et al. [94]  Italy | Population-based control survey | 59 | 44 | 12 | 32 | 0 |
| D'Amelio et al. [93]*  Italy | Epidemiological survey | 59 | 48 | 16 | 32 | 0 |
| Das et al. [95]  India | Longitudinal survey (23 PD cases) | 23 | 13 | 0 | 13 | 0 |
| Diem Zangerl et al. [96]  Austria | Prospective cohort | 238 | 189 | 7 | 182 | 0 |
| Driver et al. [97]  USA | Controlled prospective cohort study | 560 | 200 | 8 | 192 | 0 |
| Fall et al.* [98]  Sweden | Prospective | 170 | 83 | 39 | 44 | 0 |
| Gray et al. [99]  UK | Retrospective cohort study | 109 | 46 | 21 | 25 | 0 |
| Grytten et al. [100]  Norway | Longitudinal study | 878 | 198 | 10 | 175 | 13 |
| Hirst et al. [101]  UK | Prospective cohort study | 221 | 221 | 105 | 116 | 0 |
| Pennington et al. [93]  UK | Retrospective database case review | 143 | 63 | 16 | 47 | 0 |
| Schupbach et al. [102]  France | Consecutive patient chart review | 171 | 16 | 1 | 9 | 6 |
| Vergani et al. [103]  Italy | Consecutive patient chart review | 141 | 1 | 1 | 0 | 0 |
| Wilder et al. [104]  Switzerland | Longitudinal study | 50 | 17 | 0 | 15 | 2 |
| Total number (%) |  |  | 1288 | 253 (19.6) | 1014 (78.7) | 21 (1.6) |
| **Overall total number (%)** |  |  | 3909 | 952 (24.4) | 2747 (70.3) | 189 (4.8) |

**Reported in Pennington et al [93]; ALS, amyotrophic lateral sclerosis; AP, aspiration pneumonia; MS, multiple sclerosis; PD, Parkinson's disease.*
