# Supplementary material for: Dysphagia as a risk factor for mortality in Niemann-Pick disease type C: systematic literature review and evidence from studies with miglustat
Source: Orphanet J Rare Dis. 2012 Oct 6;7:76. doi: 10.1186/1750-1172-7-76 (PMC3552828; doi:10.1186/1750-1172-7-76)
Supplement: Additional file 3 — Table S3. Results for literature search to determine the prevalence of dysphagia in neurodegenerative diseases including NP-C [103-142]. [file 1750-1172-7-76-S3.doc]

**Table S3. Results for literature search to determine the prevalence of dysphagia in neurodegenerative diseases including NP-C**

| **Author / country** | **Study design** | **N** | **Dysphagia** | **No dysphagia** |
| --- | --- | --- | --- | --- |
| **ALS** |  |  |  |  |
| Amin et al. [105]  USA | Prospective case study | 22 | 11 | 11 |
| Atsuta et al. [53]  Japan | National Health and Welfare database analysis | 3428 | 723 | 2705 |
| Fattori et al. [106]  Italy | Case control study | 28 | 18 | 10 |
| Goh et al. [107]  Malaysia | Consecutive case study analysis | 73 | 21 | 52 |
| Larson and Tiryaki [108]  USA | Retrospective chart review | 58 | 31 | 27 |
| Nakayama et al. [109]  Japan | Prospective cohort study | 15 | 12 | 3 |
| Nozaki et al. [110]  Japan | Retrospective case control study | 20 | 13 | 7 |
| Rodriguez de Rivera et al. [111]  Spain | Consecutive case study analysis | 42 | 26 | 16 |
| Tomik et al. [112]  Poland | Consecutive case study analysis | 23 | 15 | 8 |
| Werneck et al. [113]  Brazil | Consecutive case study analysis | 251 | 88 | 164 |
| *Total number (%)* |  | *3960* | *958 (24.2)* | *3003 (75.8)* |
| **Huntington's chorea** |  |  |  |  |
| Aziz et al. [114]  Netherlands | Questionnaire-based study | 63 | 35 | 28 |
| Guo et al. [115]  China | Case report review | 243 | 94 | 149 |
| Guo et al. [116]  China | Case report review | 241 | 93 | 148 |
| *Total number (%)* |  | *547* | *222 (41.0)* | *325 (59.4)* |
| Bergamaschi et al. [117]  Italy | DYMUS questionnaire | 1743 | 527 | 1216 |
| Bergamaschi et al. [118]  Italy | DYMUS questionnaire | 226 | 41 | 185 |
| Poorjavad et al. [119]  Iran | Consecutive cohort analysis | 101 | 32 | 69 |
| Solaro et al. [120]  Italy | DYMUS questionnaire | 1813 | 568 | 1245 |
| Vazirinejad et al. [121]  UK | Questionnaire | 201 | 52 | 149 |
| *Total number (%)* |  | *4084* | *1220 (29.9)* | *2864 (70.1)* |
| **Neuroferritinopathy** |  |  |  |  |
| Chinnery et al. [122]  UK | Consecutive case study analysis | 41 | 16 | 25 |
| *Total number (%)* |  | *41* | *16 (39.0)* | *25 (61.0)* |
| **Neuromuscular disease** |  |  |  |  |
| Fiorenza et al. [123]  Italy | Consecutive case study analysis | 95 | 34 | 61 |
| *Total number (%)* |  | *95* | *34 (35.8)* | *61 (64.2)* |
| **NP-C** |  |  |  |  |
| Fecarotta et al. [18]  Italy | Case study analysis | 4 | 3 | 1 |
| Garver et al. [14]  USA | Questionnaire-base study | 87 | 69 | 18 |
| Patterson et al. [26]  USA | Open-label non-controlled prospective study with extension | 12 | 4 | 8 |
| Pineda et al. [70]  International | Multicentre retrospective cohort study | 63 | 51 | 12 |
| Sevin et al. [8]  France | Case study analysis | 13 | 9 | 4 |
| Wraith et al. [10]  International | Multicentre observational retrospective cohort study | 56 | 10 | 46 |
| Wraith et al. [27]  UK and USA | Non-controlled open-label extension study | 29 | 18 | 11 |
| *Total number (%)* |  | *264* | *164 (62.1)* | *100 (37.9)* |
| Althaus et al. [124]  Germany | Cross-sectional study using MADRS questionnaire | 220 | 48 | 172 |
| Choi et al. [125]  Rep. of Korea | Consecutive case study analysis | 54 | 14 | 40 |
| Coelho et al. [126]  Portugal and Spain | Consecutive cross-sectional case study analysis | 50 | 34 | 16 |
| Felix et al. [127]  Brazil | Case study analysis | 4 | 1 | 3 |
| Galazky et al. [128]  Germany | Case study analysis | 7 | 5 | 2 |
| Gonzalez-Fernandez [51]  USA | Case control study | 2933 | 249 | 2684 |
| Gross et al. [129]  USA | Prospective repeated measures study | 25 | 5 | 20 |
| Kalf et al. [130]  Netherlands | Consecutive case study analysis | 200 | 118 | 82 |
| Kelchner et al. [131]  USA | Prospective randomised multicentre trial | 711 | 533 | 178 |
| Lam et al. [132]  China | Prospective case study analysis | 45 | 6 | 39 |
| Lo et al. [133]  USA | Retrospective case study analysis | 466 | 108 | 358 |
| Manor et al. [134]  Israel | Consecutive case study analysis | 69 | 38 | 31 |
| Manor et al. [135]  Israel | Consecutive case study analysis | 57 | 41 | 16 |
| Miller et al. [136]  UK | Prospective community- and hospital-based cohort study | 137 | 44 | 93 |
| Nicaretta et al. [137]  Japan | Consecutive case study analysis | 66 | 27 | 39 |
| Nobrega et al. [138]  Brazil | Consecutive case study analysis | 19 | 4 | 15 |
| Nobrega et al. [139]  Brazil | Questionnaire-based study | 16 | 15 | 1 |
| Nobrega et al. [140]  Brazil | Case control study | 16 | 15 | 1 |
| Perez-Lloret et al. [141]  France | Cross-sectional survey | 450 | 77 | 373 |
| Rozenberg [142]  Israel | Case study analysis | 13 | 7 | 6 |
| Sung et al. [64]  Rep. of Korea | Consecutive case study analysis | 54 | 22 | 32 |
| *Total number (%)* |  | *5612* | *1411 (25.1)* | *4201 (74.9)* |
| **Progressive supranuclear palsy** |  |  |  |  |
| Warnecke et al. [143]  Germany | Consecutive case study analysis | 33 | 28 | 5 |
| *Total number (%)* |  | *33* | *28 (84.8)* | *5 (15.2)* |
| **Wilson's disease** |  |  |  |  |
| Kumar et al. [144]  India | Case study analysis | 28 | 12 | 16 |
| *Total number (%)* |  |  | *12 (42.8)* | *16 (57.2)* |
| **Overall number (%)** |  | 14664 | 4065 (27.7) | 10600 (72.3) |

ALS, amyotrophic lateral sclerosis; MS, multiple sclerosis; NP-C, Niemann-Pick disease type C; PD, Parkinson's disease
