# Supplementary material for: Dysphagia as a risk factor for mortality in Niemann-Pick disease type C: systematic literature review and evidence from studies with miglustat
Source: Orphanet J Rare Dis. 2012 Oct 6;7:76. doi: 10.1186/1750-1172-7-76 (PMC3552828; doi:10.1186/1750-1172-7-76)
Supplement: Additional file 4 — Table S4.Literature search results for the association between dysphagia and aspiration pneumonia. [file 1750-1172-7-76-S4.doc]

**Table S4. Literature search results for the association between dysphagia and aspiration pneumonia**

| **Author / country** | **Study design** | **N** | **Dysphagia** | **No dysphagia** | **Dysphagia and AP** | **Dysphagia and no AP** |
| --- | --- | --- | --- | --- | --- | --- |
| **Hospital patients** |  |  |  |  |  |  |
| Altman et al. [56]  USA | National hospital database analysis | 77,540,204 | 271,983 | 77,268,221 | 32,643 | 239,340 |
| **Stroke patients** |  |  |  |  |  |  |
| Ahn et al. [54]  Rep. of Korea | Consecutive case study analysis | 479 | 147 | 332 | 23 | 124 |
| Alshekhlee et al. [55]  USA | Retrospective singe-centre protocol | 187 | 79 | 108 | 21 | 58 |
| Aviv et al. [57]  USA | Prospective case series | 40 | 20 | 20 | 6 | 0 |
| Chua and Kong [58]  Singapore | Consecutive case study analysis | 53 | 21 | 32 | 5 | 15 |
| Daniels et al. [36]  USA | Prospective case series | 55 | 36 | 19 | 1 | 35 |
| James [59]  UK | Retrospective case note analysis | 126 | 66 | 60 | 18 | 48 |
| Meng et al. [60]  Taiwan | Retrospective medical chart review | 36 | 29 | 7 | 4 | 25 |
| Perry and Mclaren [61]  UK | Prospective case series | 185 | 77 | 108 | 15 | 62 |
| Spencer et al. [63]  USA | Prospective chart review | 102 | 73 | 29 | 13 | 60 |
| Sung et al. [64]  Rep. of Korea | Consecutive case study analysis | 294 | 65 | 224 | 18 | 47 |
| **Traumatic brain injury** |  |  |  |  |  |  |
| Schurr et al. [62]  USA | Consecutive case study analysis | 47 | 24 | 23 | 1 | 23 |

AP, aspiration pneumonia.
