# Supplementary material for: Dysphagia as a risk factor for mortality in Niemann-Pick disease type C: systematic literature review and evidence from studies with miglustat
Source: Orphanet J Rare Dis. 2012 Oct 6;7:76. doi: 10.1186/1750-1172-7-76 (PMC3552828; doi:10.1186/1750-1172-7-76)
Supplement: Additional file 5 — Table S5. Literature search results regarding the association between aspiration pneumonia and mortality. [file 1750-1172-7-76-S5.doc]

**Table S5.** **Literature search results regarding the association between aspiration pneumonia and mortality**

| **Author / country** | **Study design** | **N** | **AP** | **No AP** | **No. deaths** | **No. deaths due to AP** |
| --- | --- | --- | --- | --- | --- | --- |
| **Epilepsy** |  |  |  |  |  |  |
| Amare and Amanuel [66]  Ethiopia | Retrospective case series | 119 | 61 | 58 | 24 | 16 |
| **Stroke** |  |  |  |  |  |  |
| Ali et al. [65]  Pakistan | Prospective observational study | 100 | 28 | 72 | 7 | 4 |
| Aslanyan et al. [67]  UK | Randomised double-blind, placebo-controlled trial | 1,455 | 198 | 1,257 | 142 | 34 |
| Marwat et al. [69]  Pakistan | Descriptive case series | 93 | 28 | 65 | 7 | 3 |
| **PD** |  |  |  |  |  |  |
| Fernandez and Lapane [68]  USA | Longitudinal follow-up study | 15,186 | 101 | 15,085 | 7,614 | 76 |
| **Mixed population*** |  |  |  |  |  |  |
| Low et al. [33]  New Zealand | Consecutive case study analysis | 140 | 37 | 103 | 54 | 26 |

*Parkinson's disease, Huntington's chorea, motor-neurone disease, Alzheimer's disease.
